# Hyper-active non-homologous end joining selects for synthetic lethality resistant and pathological Fanconi anemia hematopoietic stem and progenitor cells

**DOI:** 10.1038/srep22167

**Published:** 2016-02-26

**Authors:** Wei Du, Surya Amarachintha, Andrew F. Wilson, Qishen Pang

**Affiliations:** 1 Division of Experimental Hematology and Cancer Biology, Cincinnati Children’s Hospital Medical Center, Cincinnati, Ohio 45229; 2 Department of Pediatrics, University of Cincinnati College of Medicine, Cincinnati, Ohio 45229.

## Abstract

The prominent role of Fanconi anemia (FA) proteins involves homologous recombination (HR) repair. Poly[ADP-ribose] polymerase1 (PARP1) functions in multiple cellular processes including DNA repair and PARP inhibition is an emerging targeted therapy for cancer patients deficient in HR. Here we show that PARP1 activation in hematopoietic stem and progenitor cells (HSPCs) in response to genotoxic or oxidative stress attenuates HSPC exhaustion. Mechanistically, PARP1 controls the balance between HR and non-homologous end joining (NHEJ) in double strand break (DSB) repair by preventing excessive NHEJ. Disruption of the FA core complex skews PARP1 function in DSB repair and led to hyper-active NHEJ in *Fanca*^−/−^ or *Fancc*^−/−^ HSPCs. Re-expression of PARP1 rescues the hyper-active NHEJ phenotype in *Brca1*^−/−^*Parp1*^−/−^ but less effective in *Fanca*^−/−^*Parp1*^−/−^ cells. Inhibition of NHEJ prevents myeloid/erythroid pathologies associated with synthetic lethality. Our results suggest that hyper-active NHEJ may select for “synthetic lethality” resistant and pathological HSPCs.

Fanconi anemia (FA) is a genetic disorder associated with bone marrow (BM) failure and malignancies including leukemia and solid cancers^1^,^2^,^3^,^4^. To date, eighteen known FA subtypes have been identified^5^,^6^,^7^,^8^. Patients with mutations in any of the 18 genes lead to an FA phenotype manifested by developmental abnormalities, BM failure and cancer^5^,^6^,^7^,^8^,^9^. In response to DNA damage or replicative stress, eight FA proteins (FANCA, -B, -C, -E, -F, -G, -L, and -M) form the FA core complex, which acts as an ubiquitin ligase. This FA core complex monoubiquitinates two downstream FA proteins, FANCD2 and FANCI, which then recruit other downstream FA proteins including FANCD1/BRCA2, FANCN/PALB2, FANCJ/BRIP1, FANCO/RAD51C, FANCP/SXL4, FANCQ/XPF, and possibly other DNA repair factors, to nuclear loci containing damaged DNA and consequently influence important cellular processes such as DNA replication, cell-cycle control, and DNA damage response and repair^10^,^11^.

Compelling evidence suggested that the FA pathway promotes the error-free homologous recombination (HR) repair pathway while suppressing the error-prone non-homologous end-joining (NHEJ) pathway^12^,^13^,^14^,^15^. Using FA-deficient C. elegans, chicken and human cells, two recent studies demonstrate that FA deficiency enhances the error-prone NHEJ repair, leading to increased genomic instability^12^,^15^. Genetic or pharmacological inhibition of the NHEJ rescues the FA phenotype. Another similar study also shows that inhibition of the NHEJ ligase, LIG4, ameliorates the FA phenotype, but has no effect on BRCA1 deficiency^16^. It appears the FA pathway may act to prevent inappropriate recruitment of NHEJ factors to sites of DNA damage. However, the exact mechanism by which the FA pathway counteracts the NHEJ pathway is largely unknown.

Poly (ADP-ribose) polymerase 1 (PARP1) is a major DNA damage response protein primarily involved in the base excision repair (BER) pathway^17^. Its (ADP-ribose) polymerase activity is acutely regulated by interaction with DNA breaks^17^,^18^. Upon activation at sites of DNA damage, PARP1 modifies itself and other proteins by covalent addition of long, branched polymers of ADP-ribose, which in turn recruit downstream DNA repair and chromatin remodeling factors^17^. The critical role of PARP1 in DNA repair is reflected by its frequent upregulation in cancer cells^19^,^20^, as well as the hypersensitivity of *Parp1*-deficient animals to the mutagenic effects of DNA-damaging agents^21^. Since PARP1 is involved in the repair of modified bases, single-stranded breaks (SSBs) and double-stranded break (DSB)^22^, blocking the ADP-ribosylation activity with small molecules, can achieve synthetic lethality with DNA damaging agents in the treatment of certain cancers^19^,^20^,^23^,^24^,^25^,^26^,^27^,^28^,^29^. It has been shown that PARP inhibitors could selectively target cancer cells with a defective HR repair of DSB^30^. For example, *BRCA1*-, *BRCA2*-, and *ATM*-deficient cells show hypersensitivity to PARP inhibitors, leading to genomic instability and eventual cell death due to the development of non-viable genetic errors generated by the error-prone NHEJ repair^31^,^32^,^33^,^34^.

In the current study, we show increased PARP1 activity in murine hematopoietic stem and progenitor cells (HSPCs) in response to genotoxic and oxidative stresses. Stress-induced PARP1 activation attenuates HSC exhaustion and regulates HR-NHEJ balance in DSB repair. Using mice deleted for the FA core complex genes encoding *Fanca* and *Fancc*, we demonstrate that PARP1 coordinates with the FA pathway to prevent excessive NHEJ in stressed HSPCs. Furthermore, we employed two lines of synthetic-lethality murine models to show that hyper-active NHEJ resulting from synthetic lethality selects for resistant and pathological HSPCs and that inhibition of NHEJ ameliorates hematological pathologies associated with synthetic lethality.

## Results

### PARP1 activation in HSPCs in response to genotoxic and oxidative stresses

**Figure 1:**
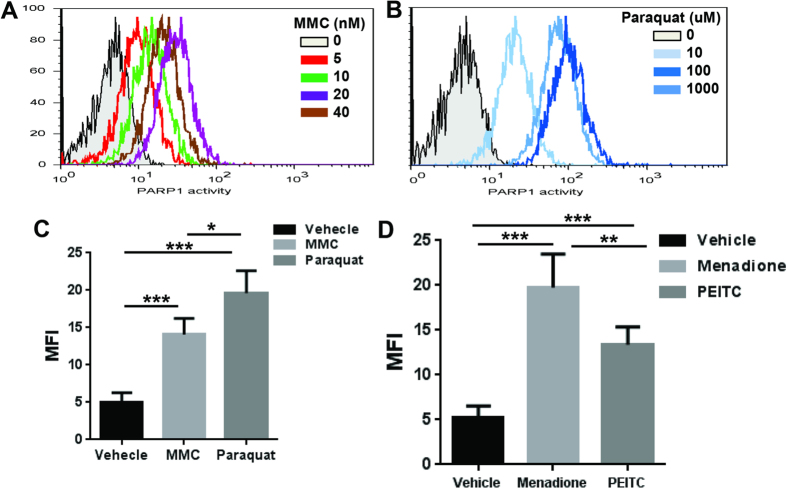
PARP1 activation in HSPCs in response to genotoxic and oxidative stress. (**A**) PARP1 activation in HSPCs in response to MMC. Lin^−^Sca1^+^c-kit^+^ (LSK) cells isolated from Wild-type (WT) mice were treated with the indicated doses of MMC in StemSpan medium supplemented with 50 ng/ml murine rTpo, 50 ng/ml murine rSCF and 1% BSA for 12 hours, followed by Flow cytometric analysis for PARP1 activity. (**B**) PARP1 activation in HSPCs in response to oxidative stress. Cells described in (**A**) were cultured in the presence of the indicated doses of paraquat for 4 hours. Cells were then subjected to Flow cytometric analysis for PARP1 activity. (**C**) PARP1 activation in MEFs in response to MMC and paraqaut. Mouse embryonic fibroblasts (MEFs) isolated from WT mice were treated with MMC (20 nM) for 12 hours or paraquat (100 μM) for 4 hours followed by Flow cytometric analysis for PARP1 activity. (**D**) PARP1 activation in HSPCs in response to endogenous oxidative stress. Cells described in (**A**) were treated with either 100 μM menadione for 4 hours or 10 μM PEITC for 6 hours followed by Flow cytometric analysis for PARP1 activity. Results are means ± standard deviation (SD) of three independent experiments.

The hematopoietic system is particularly vulnerable to various insults, including genotoxic and oxidative stress^35^. To understand the function of PARP1 in stressed hematopoiesis, we first measured PARP1 activity in HSPCs in response to genotoxic stress. We isolated BM LSK (Lin^−^c-kit^+^Sca-1^+^) cells from WT C57BL/6 mice using the gating strategy shown in Fig. S1A. We treated the FACS-sorted LSK cells with increasing doses of mitomycin C (MMC), a DNA cross-linking agent, for 12 hours followed by Flow cytometric analysis for PARP1 activity. MMC treatment led to a dose-dependent increase in PARP1 activation, which peaked at 20 nM of MMC (Fig. 1A). We also determined the kinetics of PARP1 activation in response to the genotoxic insult by treating LSK cells with 20 nM MMC for different time intervals. We observed that PARP1 activation peaked at 12 hours and returned to baseline level by 24 hours after MMC treatment (Fig. S1B).

We next examined PARP1 activation in HSPCs in response to oxidative stress. By treating BM LSK cells with Paraquat (PA), an agent that induces accumulation of reactive oxygen species (ROS)^36^, we observed a dose-dependent increase in PARP1 activation in LSK cells with a peak at 100 μM of Paraquat (Fig. 1B). Paraquat-induced PARP1 activation peaked at 4 hours and declined to basal level by 8 hours (Fig. S1B). Similar results were obtained with primary murine embryonic fibroblast (MEF) cells (Fig. 1C). Thus, PARP1 is activated in HSPCs in response to both genotoxic and oxidative stresses.

It is known that even in physiological state, HSPCs are exposed to various ROS^37^,^38^,^39^. To determine if endogenous oxidative stress induced PARP1 activation in HSPCs, we treated BM LSK cells with the mitochondrial uncoupler menadione or PEITC, which depletes glutathione and induces endogenous oxidative stress^40^,^41^. We observed significantly increased PARP1 activation with either 100 μM menadione or 10 μM PEITC (Fig. 1D). Pre-treatment of the cells with the ROS scavenger N-acetyl cysteine (NAC; 100 μM) or catalase (100 μg/ml) reduced menadione or PEITC-induced PARP1 activation (Fig. S1C). These results indicate that both exogenous and endogenous ROS can induce PARP1 activation in HSPCs.

We next analyzed apoptosis induced by MMC and Paraquat treatments using Annexin V staining. While we observed a dose-dependent increase of apoptosis in MMC- and Paraquat-treated LSK cells, the percentages of the apoptotic cells treated with 20 nM MMC for 12 h or 100 μM Paraquat for 4 h were less than 10% (Fig. S1D), indicating insignificant induction of apoptosis by both agents at their respective peak doses and optimal timing of PARP1 activation.

### PARP1 activation attenuates oxidative stress-induced HSC exhaustion

**Figure 2:**
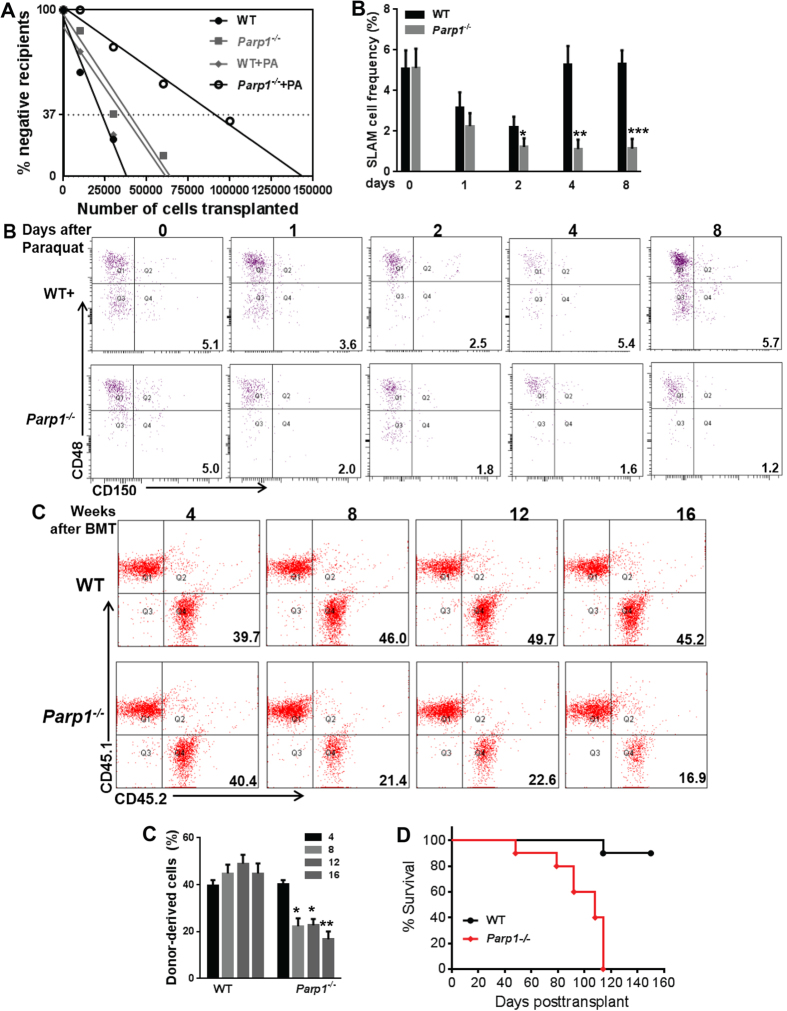
PARP1 activation attenuates Oxidative stress-induced HSC exhaustion. (**A**) Analysis of oxidative stress-induced HSC exhaustion by limiting dilution assay. Low density bone marrow cells (LDBMCs) from WT or *Parp1*^−/−^ mice were treated with or without paraquat (100 μM) *ex vivo* for 4 h. Graded numbers of the treated cells plus 2 × 10^5^ radio-protector BM cells were then transplanted to lethally irradiated recipients. Plotted are the percentages of recipients containing less than 1% donor (CD45.2^+^) blood nucleated cells at 16 weeks post-transplantation. Frequency of functional HSCs was calculated according to Poisson statistics. WT: 1/24,768 (p = 0.1353); *Parp1*^−/−^: 1/39,736 (p = 0.0658); WT+PA: 1/36,063 (p = 0.1083); *Parp1*^−/−^+PA: 1/91,333 (*p* = 0.001). (**B**) PARP1 prevents oxidative stress-induced HSC reduction *in vivo*. Single dose of paraquat (10 mg/kg) was administrated to WT or *Parp1*^−/−^ mice. LDBMCs were then isolated at the indicated time points and analyzed for frequency of HSCs (SLAM: Lin^−^Sca1^+^c-kit^+^CD150^+^CD48^−^ cells) by Flow Cytometry. Representative dot plots and quantification are shown. Results are means ± standard deviation (SD) of three independent experiments. (**C**) *Parp1* deficiency compromises repopulating capacity of HSCs under Paraquat-mediated oxidative stress. 50 SLAM cells from the mice described in (**B**) along with 2 × 10^5^ radio-protective cells were transplanted to lethally irradiated recipients. Donor-derived chimera were assessed 4, 8, 12, 16 weeks post BMT. Representative dot plots and quantification are shown. Results are means ± standard deviation (SD) of 3 independent experiments (n = 9 per group). (**D**) Survival of the secondary recipient mice. One million LDBMCs from mice described in (**B**) were transplanted into sublethally (6 Gy) irradiated primary recipients. 4 months after primary transplantation, 1 × 10^6^ BM cells from the primary recipients were further transplanted into lethally (8 Gy) irradiated secondary recipients (n = 10 per group). Survival of the recipients is plotted by the Kaplan-Meier curve method and analyzed by the log-rank test.

We and others have shown that oxidative stress is a major physiological driver in HSC exhaustion^37^,^38^,^39^. Because PARP1 is activated in HSPCs in response to both exogenous and endogenous ROS, we next performed three *in vivo* experiments to determine the role of PARP1 in HSC maintenance. First, we carried out a limiting dilution assay^42^, in which we treated low density BM cells (LDBMCs) from WT or *Parp1*^−/−^ mice *ex vivo* with 100 μM of Paraquat. We then transplanted graded numbers of these treated cells, along with 2 × 10^5^ radio-protector BM cells, into lethally irradiated congenic recipients, and analyzed the frequency of functional HSCs in the tested LDBMCs. 16 weeks later, *Parp1* deficiency demonstrated a 2.5-fold reduction in the frequency of long-term (LT)-HSCs (1 in 91,333 LDBMCs in Paraquat-treated *Parp1*^−/−^ donors and 1 in 36,063 LDBMCs in Paraquat-treated WT donors; Fig. 2A).

Second, we injected WT and *Parp1*^−/−^ mice with a single dose of paraquat (10 mg/kg) and analyzed the frequency of phenotypic LT-HSCs (Lin^−^ckit^+^Sca-1^+^CD150^+^CD48^−^; SLAM)^43^,^44^ at different time points after injection (Fig. S2A). Paraquat significantly reduced SLAM cell frequency in both WT and *Parp1*^−/−^ mice during the first two days following injection (Fig. 2B). However, WT mice underwent complete HSC recovery on day 4 post injection. In contrast, HSC recovery was not observed in paraquat-treated *Parp1*^−/−^ mice during the 8-day period post-paraquat injection (Fig. 2B). It is noteworthy that paraquat-induced HSC depletion was much more profound in *Parp1*^−/−^ mice than in their WT littermates. The failure in HSC recovery observed in *Parp1*^−/−^ mice was not due to increased apoptosis (Fig. S2B). However, paraquat treatment increased accumulation of G_1_ and S/G_2_/M cells at the expense of quiescent (G_0_) cells in both WT and *Parp1*^−/−^ mice (Fig. S2C).

Third, we performed BM transplantation (BMT) by injecting 50 SLAM cells from paraquat-treated WT and *Parp1*^−/−^ mice, along with 2 × 10^5^ radio-protective recipient cells, into lethally irradiated recipients and monitored donor-derived hematopoiesis at different time points post BMT. We observed sustained donor reconstitution (40–50%) in the recipient mice transplanted with WT cells up to 16 weeks after BMT, indicating long-term hematopoiesis (Fig. 2C). However, in the recipients transplanted with *Parp1*^−/−^ cells, donor-derived chimerism was robustly established at 4 weeks post BMT but declined progressively afterwards (40.5 ± 5.58% at 4 weeks vs 20.5 ± 4.44% at 12 weeks and 15.7 ± 3.15% at 16 weeks; Fig. 2C). These results indicate that *Parp1*^−/−^ HSCs underwent replicative exhaustion *in vivo* under oxidative stress. Furthermore, serial BMT experiments show that all 10 secondary recipients of *Parp1*^−/−^ cells died within 4 months post-transplantation, while majority of the recipients of WT cells survived beyond 5 months (Fig. 2D). Taken together, PARP1 activation attenuates oxidative stress-induced HSC exhaustion.

### PARP1 regulates HR-NHEJ balance in DSB repair

**Figure 3:**
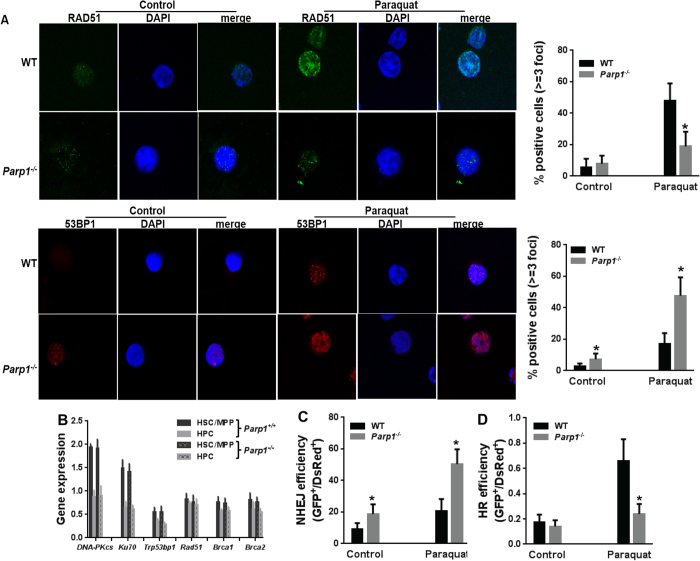
PARP1 regulates HR-NHEJ balance in DSB repair. (**A**) PARP1 is essential for preventing excessive NHEJ. LSK cells isolated from *Parp1*^+/+^ or *Parp1*^−/−^ mice were treated with 100 μM of paraquat for 2 hours then release in fresh medium for 24 hours *in vitro*. Cells were then subjected to immunofluorescence staining using antibodies against Rad51 (Upper), or 53BP1 (Lower). Representative images (Left) and quantification (Right) are shown. Results are means ± standard deviation (SD) of 3 independent experiments. (**B**) Higher expression of NHEJ genes in HSC/MPP than HPC cells. RNA was extracted from HSC/MPP (Lin^−^ckit^+^Sca-1^+^CD150^+^CD48^−^ and Lin^−^ckit^+^Sca-1^+^CD150^−^CD48^−^) and HPC (Lin^−^ckit^+^Sca-1^+^CD150^−^CD48^+^ and Lin^−^ckit^+^Sca-1^+^CD150^+^CD48^+^) cells isolated from WT mice followed by RT-PCR analysis for *DNA-PKcs, Ku70, Trp53bp1, Rad51, Brca1,* and *Brca2* expression. Samples were normalized to the level of *GAPDH* mRNA. (**C**) Deletion of *Parp1* leads to increased NHEJ efficiency. Mouse embryonic fibroblasts (MEFs) from WT or *Parp1*^−/−^ mice were transfected with retroviral vectors expressing NHEJ-eGFP and DsRed followed by *in vitro* culture with paraquat. Repair efficiency was determined by the ratio of eGFP to DsRed. (**D**) PARP1 stimulated HR. MEF cells from WT or *Parp1*^−/−^ mice were transfected with retroviral vector expressing HR-eGFP and DsRed followed by *in vitro* culture with paraquat. Repair efficiency was determined by the ratio of eGFP to DsRed. Results are means ± standard deviation (SD) of 3 independent experiments.

We described above that MMC and oxidative stress induced robust PARP1 activation in HSPCs (Fig. 1). Since both MMC and ROS generate DSBs, which can be repaired by HR and NHEJ in a balanced and cell-context manner^45^ we used two established assays to determine the potential role of PARP1 in regulating HR-NHEJ balance. First, we analyzed the repair in paraquat-treated WT and *Parp1*^−/−^ LSK cells using 53BP1- and RAD51-foci formation as the surrogates for NHEJ and HR^46^, respectively. We found that in WT cells, paraquat induced robust RAD51 foci formation with approximately 50% of the cells containing 3 or more RAD51 foci (Fig. 3A upper). On the other hand, *Parp1*^−/−^ LSK cells showed predominantly NHEJ activity, as evidenced by ~60% of the cells with 3 or more 53BP1 foci (Fig. 3A lower). To rule out the possibility of differential expression of the genes involved in HR or NHEJ in WT and *Parp1*^−/−^ cells, we compared the expression of several key HR (*Rad51, Brca1, Brca2*) and NHEJ (*DNA-PKcs, Ku70, Trp53bp1*) genes in two HSPC subsets: the primitive HSC/MPP (Lin^−^ckit^+^Sca-1^+^CD150^+^ CD48^−^ and Lin^−^ckit^+^Sca-1^+^CD150^−^CD48^−^) and the more mature progenitor HPC (Lin^−^ckit^+^Sca-1^+^CD150^−^CD48^+^ and Lin^−^ckit^+^Sca-1^+^CD150^+^CD48^+^) populations. Consistent with previous report^44^, we found higher expression of NHEJ genes in HSC/MPP cells than in HPC cells (Fig. 3B). However, no differential expression of these genes was found between WT and *Parp1*^−/−^ cells.

Second, we employed an enhanced green fluorescent protein (eGFP)-based reporter system^47^, in which induction of DSB by I-SceI inactivates the eGFP gene, to examine the preferential utilization of HR or NHEJ repair pathway in WT and *Parp1*^−/−^ cells. Due to inefficient transfection in LSK cells, we chose to conduct this assay in primary WT and *Parp1*^−/−^ MEF cells by DNA transfection. To exclude potential transfection difference, pDsRed2-N1 plasmid was used as control. By comparing the ratio between eGFP and DsRed (Fig. S3), we were able to demonstrated that the efficiency of NHEJ in *Parp1*^−/−^ MEF cells was much greater than that in WT cells in response to paraquat treatment (Fig. 3C). On the other hand, the usage of HR is significantly higher in WT cells upon oxidative stress (Fig. 3D), suggesting a crucial role of PARP1 in promoting HR.

### FA deficiency skews HR-NHEJ balance regulated by Parp1 in DSB repair

**Figure 4:**
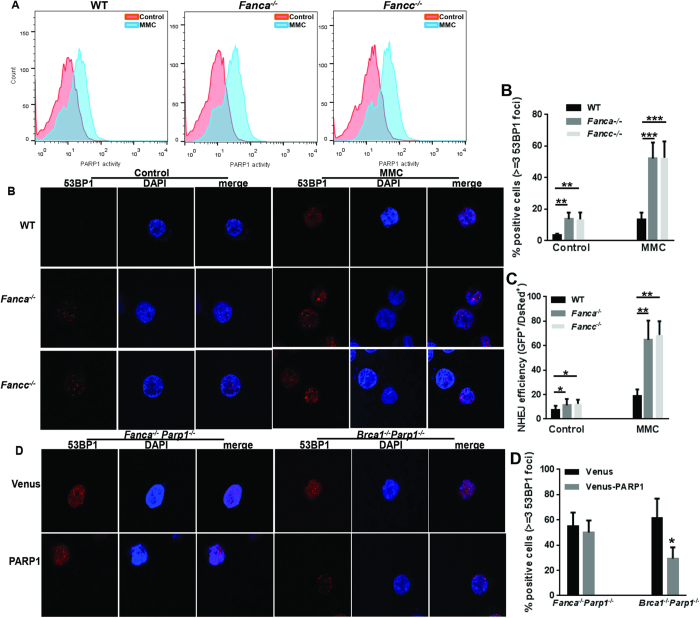
FA deficiency skews Parp1 function in DSB repair. (**A**) MMC-induced PARP1 activation is preserved in *Fanca*^−/−^ or *Fancc*^−/−^ HSCs. LDBMCs from WT, *Fanca*^−/−^ or *Fancc*^−/−^ mice were treated with 20 nM MMC for 12 hours followed by Flow Cytometric analysis for PARP1 activity in LSK cells. (**B**) Inactivation of *Fanca* or *Fancc* enhances DNA damage-induced NHEJ in HSPCs. LSK cells isolated from WT, *Fanca*^−/−^, or *Fancc*^−/−^ mice were treated in MMC (20 nM) for 12 hours followed by immunofluorescence staining using antibodies against 53BP1. Representative images (Upper) and quantification (Lower) were shown. (**C**) Inactivation of *Fanca* or *Fancc* enhances DNA damage-induced NHEJ in MEFs. MEF cells isolated from WT, *Fanca*^−/−^, or *Fancc*^−/−^ mice were transfected with plasmids expressing NHEJ-eGFP and DsRed followed by *in vitro* culture with MMC (20 nM) for 12 hours. Repair efficiency was determined by the ratio of eGFP to DsRed. (**D**) Re-expression of PARP1 does not rescue hyper-active NHEJ in *Fanca*^−/−^*Parp1*^−/−^ cells. LSK cells from *Brca1*^−/−^*Parp1*^−/−^ *or Fanca*^−/−^*Parp1*^−/−^ mice were transduced with lentiviral vector expressing Venus or Venus-PARP1. Venus^+^ cells were then treated with 20 nM MMC for 12 hours followed by immunofluorescence staining using antibodies against 53BP1. Representative images (Left) and quantification (Right) were shown.

Because the FA pathway also plays a role in promoting HR while suppressing NHEJ^14^,^45^, we asked whether the FA pathway and PARP1 functionally co-operated in HSPCs during DSB repair. We first established that genotoxic stress-induced PARP1 activation was preserved in FA deficient HSPCs, as the levels of MMC-induced PARP1 activation in LSK cells deficient for the FA core complex component Fanca (*Fanca*^−/−^) or Fancc (*Fancc*^−/−^) were similar to those in WT LSK cells (Fig. 4A). Similar results were obtained with paraquat-treated cells, in which oxidative stress-induced PARP1 activation was preserved in *Fanca*^−/−^ or *Fancc*^−/−^ LSK cells (Fig. S4A). However, inactivation of *Fanca* or *Fancc* significantly increased MMC-induced 53BP1 foci (Fig. 4B), indicative of augmented NHEJ repair activity in *Fanca*^−/−^ or *Fancc*^−/−^ HSPCs. Consistently, we observed significantly higher efficiency of NHEJ in *Fanca*^−/−^ or *Fancc*^−/−^ MEF cells than in WT cells (Fig. 4C).

To examine whether the FA pathway and PARP1 co-operated in modulating the HR-NHEJ balance in DSB repair, we generated *Fanca*^−/−^*Parp1*^−/−^ double knockout (DKO) mice. We also created another DKO strain by crossing the *Parp1*^−/−^ mice with a conditional allele of *Brca1*, which is a *bona-fide* HR gene. The reason for using the *Brca1*^−/−^*Parp1*^−/−^ DKO cells was based on the observations that inhibition of PARP1 in *BRCA1*-deficient cancer cells would greatly boost NHEJ activity^32^,^33^. We hypothesized that if the FA pathway coordinated with PARP1 in regulating the HR-NHEJ balance, re-expression of PARP1 in *Fanca*^−/−^*Parp1*^−/−^ DKO cells would not be able to rescue the hyper-active NHEJ phenotype. BM LSK cells isolated from these two lines of DKO mice were transduced with lentivirus expressing Venus alone or Venus plus PARP1 (Fig. S4B). We observed a robust 53BP1 foci formation in Venus-transduced *Fanca*^−/−^*Parp1*^−/−^ and *Brca1*^−/−^*Parp1*^−/−^ LSK cells (Fig. 4D). Re-expression of PARP1 significantly reduced the cells stained positive for 53BP1 foci in *Brca1*^−/−^*Parp1*^−/−^ LSK cells; whereas this reduction was only marginal at best in *Fanca*^−/−^*Parp1*^−/−^ cells (Fig. 4D). Similar results were obtained in a repair assay using primary MEF cells from the *Fanca*^−/−^*Parp1*^−/−^ and *Brca1*^−/−^*Parp1*^−/−^ DKO mice, in which re-expression of PARP1 almost completely rescued the hyper-active NHEJ phenotype in *Brca1*^−/−^*Parp1*^−/−^ but only partially in *Fanca*^−/−^*Parp1*^−/−^ cells (Fig. S4C). These results indicate that PARP1 is inefficient in suppressing NHEJ without the FA core complex, and suggest that the FA pathway may cooperate with PARP1 in suppressing aberrant activation of the NHEJ pathway.

### Hyper-active NHEJ selects for synthetic lethality resistant HSPCs

**Figure 5:**
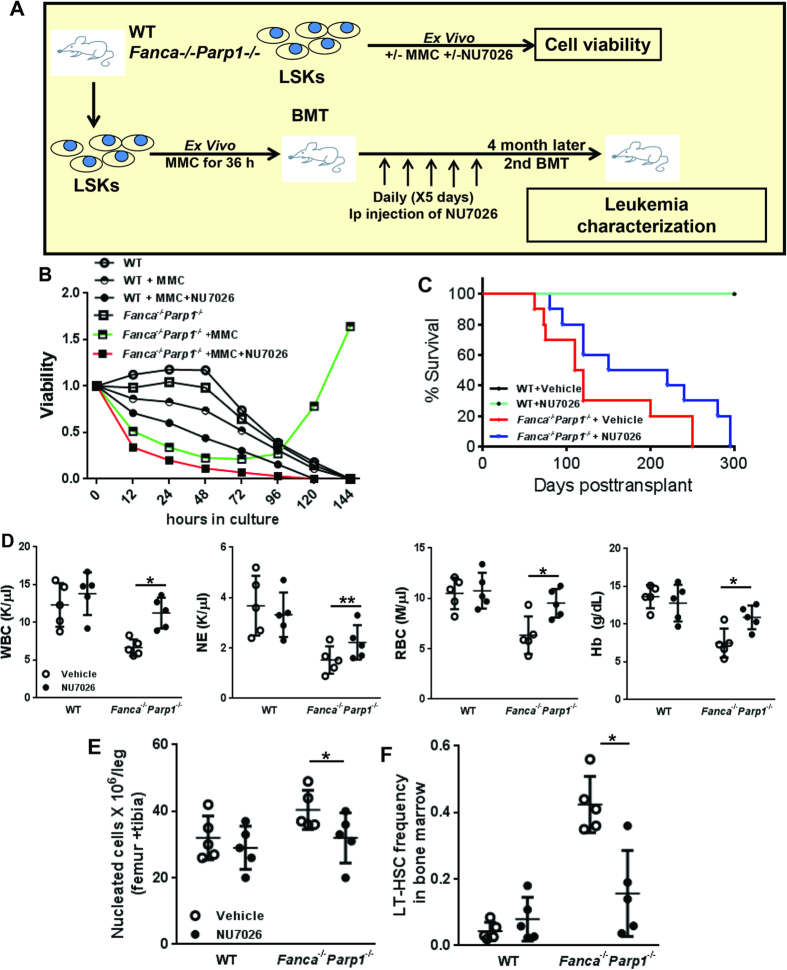
Hyper-active NHEJ selects for “synthetic lethality” resistant HSPCs. (**A**) Schematic presentation of experiment design. (**B**) DNA-PKcs inhibitor prevents the emerge of resistant *Fanca*^−/−^*Parp1*^−/−^ DKO HSPCs. LSK cells isolated from WT, *Fanca*^−/−^*Parp1*^−/−^ *or Brca1*^−/−^*Parp1*^−/−^ mice were treated with 20 nM MMC. NU7026 was added at the 36-h time point during MMC treatment. Cell viability was determined by trypan blue assay at the indicated time points. Percentages of viable cells were normalized to the numbers at day 0. (**C**) Survival of transplant recipients. LSK cells from either WT or *Fanca*^−/−^*Parp1*^−/−^ DKO mice were treated with MMC *ex vivo* for 36 hours. 3,000 viable cells along with 2 × 10^5^ c-kit depleted protector BM cells were used for BMT into lethally irradiated BoyJ recipients (n = 10 per group). NU7026 was then administrated to the recipients daily for 5 days. Survival of the recipients plotted by the Kaplan-Meier curve method and analyzed by the log-rank test. (**D**) Peripheral blood counts of the recipients. (**E,F**) BM cellularity and phenotypic HSCs in the BM of the recipients.

Inhibition of PARP1 in *BRCA1*- or *BRCA2*-deficient cancer cells leads to synthetic lethality, because these mutant cells rely on PARP1 for blocking cellular accumulation of catastrophic DSBs^31^,^32^,^33^,^34^. We thus asked whether inactivation of PARP1 in FA-deficient HSPCs could also induce synthetic lethality. We treated WT and *Fanca*^−/−^*Parp1*^−/−^ DKO LSK cells with or without MMC and determined the kinetics of cell survival (Fig. 5A). To our surprise, simultaneous deletion of *Fanca* and *Parp1* caused only marginal decrease in viability of HSPCs in the absence of MMC (Fig. 5B). However, MMC induced significant increase in cell death of the *Fanca*^−/−^*Parp1*^−/−^ DKO cells compared to WT cells, indicating an enhanced synthetic lethality in response to DSB DNA damage. Interestingly, the percentage of viable *Fanca*^−/−^*Parp1*^−/−^ DKO cells not only stopped declining but also rapidly increased after 48 h in MMC-supplemented medium; whereas the WT cells continued dying (Fig. 5B). We postulated that hyper-activated NHEJ might select for resistant *Fanca*^−/−^*Parp1*^−/−^ DKO HSPCs. To test this notion, we added a NHEJ inhibitor, NU7026 that targets DNA-PKcs^47^, to the cultures at the 36-h time-point during MMC treatment (Fig. 5A). Strikingly, we observed a complete prevention of the emergence of resistant *Fanca*^−/−^*Parp1*^−/−^ DKO cells by NU7026 (Fig. 5B). To confirm this observation *in vivo*, we treated WT and *Fanca*^−/−^*Parp1*^−/−^ DKO BM LSK cells with MMC *ex vivo* for 36 h, and performed BM transplantation by transplanting 3,000 viable cells, along with 2 × 10^5^ c-Kit-depleted protector cells, into each of lethally irradiated recipients followed by daily injection of NU7026 or vehicle for five days (Fig. 5A).

All NU7026- and vehicle-injected recipients transplanted with *Fanca*^−/−^*Parp1*^−/−^ DKO cells succumbed to bone marrow failure in less than 300 days post-transplant (Fig. 5C). We found no mortality in recipients of WT cells, either NU7026- or vehicle-injected, within 350 days post-transplant. Analysis of peripheral blood and BM of the moribund mice showed peripheral neutropenia and anemia in both NU7026- and vehicle-injected samples (Fig. 5D). However, vehicle-injected recipients of *Fanca*^−/−^*Parp1*^−/−^ DKO cells exhibited BM hypercellularity (Fig. 5E), and increased accumulation of phenotypic HSCs in the BM (Fig. 5F). NU7026 treatment greatly ameliorated all these phenotypes. No overt leukemia was observed in both NU7026- and vehicle-injected mice. Taken together, these results indicate that inhibition of NHEJ prevents myeloid and erythroid pathologies associated with synthetic lethality in *Fanca*^−/−^*Parp1*^−/−^ DKO HSPCs and suggest that hyper-active NHEJ may select for synthetic lethality resistant and pathological HSPCs, which were pre-leukemic in nature (see below).

### The synthetic lethality-resistant HSPCs cause leukemia in secondary recipients

**Figure 6:**
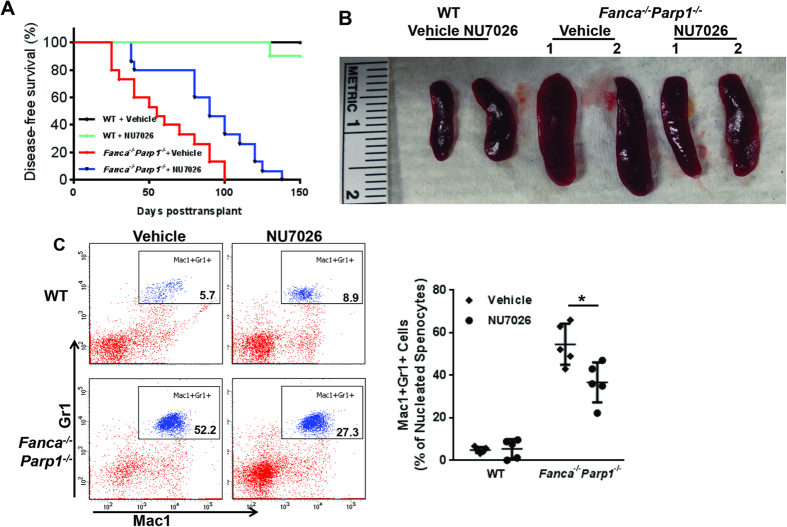
Inhibition of NHEJ delays leukemia induced by synthetic lethality-resistant HSPCs. (**A**) The synthetic lethality-resistant HSPCs cause leukemia in secondary recipients. LSK cells from WT or *Fanca*^−/−^*Parp1*^−/−^ DKO mice were treated with MMC *ex vivo* for 36 hours, and 3000 viable cells were transplanted into sublethally (6 Gy) irradiated BoyJ recipients. NU7026 was then administrated to the recipients daily for 5 days. Mice were sacrificed four months post-transplant and 3–5 million whole bone marrow cells from primary recipients were transplanted to lethally irradiated (8 Gy) 2^nd^ recipients (n = 10–15 per group). Survival of the recipients plotted by the Kaplan-Meier curve method and analyzed by the log-rank test. (**B**) Leukemic mice exhibit splenomegaly. Spleen images of moribund mice. (**C**) DNA-PK inhibition ameliorates myeloid infiltration of leukemic mice. LDBMCs from the leukemic mice were subjected to Flow Cytometric analysis for Gr1 and Mac1. Representative images (Left) and quantification (Right) were shown.

We next performed serial BMT experiments to determine if hyper-active NHEJ would ultimately predispose the resistant *Fanca*^−/−^*Parp1*^−/−^ HSPCs to leukemia. We found that 12 of 15 secondary recipients of BM cells from vehicle-injected primary *Fanca*^−/−^*Parp1*^−/−^ mice died of leukemia within 4 months (Fig. 6A; median survival 52.5 days). All the moribund mice showed splenomegaly (Fig. 6B) with myeloid infiltration (Fig. 6C). Significantly, secondary recipients of BM cells from NU7026-injected primary *Fanca*^−/−^*Parp1*^−/−^ mice also developed leukemia but with much longer latency than those transplanted with cells from the vehicle-injected primary *Fanca*^−/−^*Parp1*^−/−^ mice (median survival 117.5 days). These data indicate that the synthetic lethality-resistant HSPCs could establish disease upon secondary transfer and suggest that inhibition of NHEJ could reduce leukemia burden.

## Discussion

It has been postulated that the FA pathway suppresses NHEJ in favor of HR during DSB repair^12^,^13^,^14^,^15^. However, mechanistic underpin for this phenomenon is still lacking. In the present study, we demonstrate that the FA pathway is required for PARP1 function in regulation of HR-NHEJ balance in DSB repair and inhibition of NHEJ prevents myeloid and erythroid pathologies in the context of synthetic lethality. We also show that hyper-active NHEJ can select for synthetic lethality resistant HSPCs leading to leukemia development. To our knowledge, this is the first study that provides mechanistic insight to drug resistance generated by hyper-active NHEJ resulting from FA-PARP synthetic lethality. Furthermore, our results suggest that inhibition of NHEJ may be beneficial for FA cancer treatment in the context of PARP inhibitor therapy.

DSBs can be generated as an intermediate of normal cellular processes, such as DNA replication. Cells have evolved two major specialized mechanisms to cope with these highly deleterious lesions, namely HR and NHEJ in a balanced and cell-context manner^45^. NHEJ is error-prone DSB repair, which contributes to cancer during breakage-fusion-bridge cycles^48^,^49^. PARP1 is a major DNA damage response protein primarily involved in the BER pathway^17^. Inhibition of the PARP enzymes including PARP1 in *BRCA*-deficient cancer cells causes synthetic lethality, a phenomenon that has been successfully utilize to treat certain breast cancers^19^,^20^. We employed two FA mouse models (*Fanca* and *Fancc*) to establish FA-PARP synthetic lethality under genotoxic stress. Our study corroborates the previous observations that deficiency in non-BRCA HR proteins including FANCA, FANCC, or FANCD2 can also constitute synthetic lethality with PARP inhibition^50^. The molecular basis for HR-PARP synthetic lethality is that PARP inhibition enhances error-prone NHEJ repair, leading to genomic instability and cell death^31^,^32^,^33^,^34^. Recently, a few studies have implicated PARP1 in NHEJ. For instance, it has been proposed that PARP1 is a component of an alternative pathway of NHEJ^51^; whereas two studies using chicken DT40 cell lines shows that inactivation of NHEJ could attenuate the hypersensitivity of PARP1-deficient cells to camptothecin-generated DSBs^52^,^53^. Although these studies did not provide mechanistic detail on how PARP1 suppresses NHEJ, they suggest a potential role of PARP1 in regulating the choice of HR or NHEJ pathway for DSB repair. Our present study employed genetic models of BRCA-PARP1 and FA-PARP1 “synthetic lethality” to provide mechanistic evidence that PARP1 controls the balance between HR and NHEJ during DSB repair, possibly through preventing excessive NHEJ and facilitating HR.

It has been shown that the FA pathway plays important role in promoting HR while suppressing NHEJ^12^,^13^,^14^,^15^. Indeed, recent reports have demonstrated that FA deficiency enhances the error-prone NHEJ repair, leading to increased genomic instability. Genetic or pharmacological inhibition of the NHEJ reduces genomic instability in FA cells^12^,^15^. Another similar study also shows that inhibition of the NHEJ ligase, LIG4, ameliorates the FA phenotype, but has no effect on BRCA1 deficiency^16^. We hypothesized that the FA pathway and PARP1 might functionally co-operate in regulating the HR-NHEJ balance in DSB repair. In support of this, we employed a PARP1 reconstitution of primary cells isolated from the *Fanca*^−/−^*Parp1*^−/−^ and *Brca1*^−/−^*Parp1*^−/−^ DKO mice to demonstrate that PARP1 is inefficient in suppressing NHEJ without the FA core complex. Our findings suggest a fundamental mechanistic difference in “synthetic lethality” between FA cells and cells deficient in other genes involved in the HR repair pathway, such as *BRCA1*-, *BRCA2*-, and *ATM*^30^,^31^,^32^,^33^,^34^, whose hypersensitivity to PARP inhibition is totally dependent on suppression of NHEJ by PARP1. Therefore, our data highlights a crucial role of the FA core complex in the regulation of HR-NHEJ.

Maintenance of genome stability in the HSC compartment is essential for the sustainment of normal hematopoiesis. Numerous studies have shown that HSCs are more sensitive than other cell types and tissues to DNA damage particularly DSBs, mostly due to intrinsic limitations in DNA repair capacity^54^. In addition, it is known that quiescent human HSPCs preferentially use the NHEJ pathway to repair DSBs^55^. Consistently, we found higher expression of major NHEJ genes in primitive HSCs than more mature HPCs, indicating that HSCs might preferentially use error-prone pathway for DSB repair, which may lead to high risk of pathological transformation. We used 53BP1-foci formation as the readout for NHEJ repair efficiency, due to technical difficulty in LSK cells. We note that 53BP1-foci formation is not necessary the exclusive marker for NHEJ repair, as it has been well established that ultrafine DNA bridges (UFBs) during the procession of DSBs can induce the formation of 53BP1-foci or bodies^56^. Interestingly, the hyper-active NHEJ identified as the mechanism of resistance to “FA-PARP1 synthetic lethality” in our study is also distinct from the mechanism of resistance to PARP inhibitor-based therapy, which has thus far been reported to be mainly based on an enhanced HR repair pathway^57^ or acquired new mutations in the *BRCA2* gene that restore the full length *BRCA2* gene as well as BRCA2 function^58^,^59^. The exact mechanism by which hyper-active NHEJ induces resistance to PARP inhibitor-based therapy remains to be investigated.

## Material and Methods:

### Mice and treatment

*Fanca*^+/+^, *Fanca*^−/−^ and *Fancc*^+/+^, *Fancc*^−/−^ mice were generated by interbreeding the heterozygous *Fanca*^+/−^ (Dr. Madeleine Carreau at Laval University) or *Fancc*^+/−^ mice (Dr. Manuel Buchwald, University of Toronto, Ontario, Canada), respectively. *129SParp1*^*tm1zqw/J*^ (The Jackson Laboratory, Bar Harbor, ME) was backcrossing with WT C57BL/6 mice for eight generations before interbreeding heterozygous *Parp1*^+/−^ mice to generate *Parp1*^−/−^ mice. *Brca1 exon11* conditional deletion mice were previously described^60^. For Cre-mediated gene deletion, animals were injected i.p. with 100 ml of tamoxifen (20 mg/ml; Sigma-Aldrich, St. Louis, MO). All the animals including BoyJ mice were maintained in the animal barrier facility at Cincinnati Children’s Hospital Medical Center. All animal experiments were performed in accordance with the institutional guidelines and approved by the Institutional Animal Care and Use Committee of Cincinnati Children’s Hospital Medical Center (IACUC2013-0159).

For paraquat treatment, mice were intraperitoneal (i.p.) injected with single dose of 10 mg/kg paraquat (Sigma-Aldrich, St. Louis, MO). NU7026 (Sigma-Aldrich, St. Louis, MO) administration was conducted by intraperitoneal (i.p.) injecting 20 mg/kg of NU7026^61^ to the recipients daily for consecutive 5 days.

### DSB repair assays and FACS analysis

DSB repair assays were adapted from previously study^47^ using NHEJ reporter cassette or HR reporter cassette kindly provided by Dr. Gorbunova V at University of Rochester. Briefly, MEF cells from WT or *Parp1*^−/−^ mice were transfected with 0.5 μg of linearized NEHJ or HR reporter constructs followed by G418 selection. MEF cells containing chromosomally integrated GFP-based reporter construct were then co-transfected with 0.5 μg of plasmid encoding I-SceI endonuclease to induce DSBs. To exclude the potential transfection difference, 0.1 μg of plasmid encoding DsRed (pDsRed2-N1) was used as a control. Four days after transfection, the ratio between GFP^+^ and DsRed^+^ cells was determined by Flow cytometry as a measure of DSB repair efficiency using a FACS canto machine (BD Biosciences, San Jose, CA). For each treatment, a minimum of 20,000 cells were analyzed by FACS. Data analysis was done using FACSDiva software (BD Biosciences, San Jose, CA).

### Immunofluorescence staining

CD34^−^LSK cells from indicated mice were cytospan onto cover slips followed by fixation in 2% paraformaldehyde/PBS. Cells were then washed with PBS twice and incubated with 0.2% Triton X-100 for permeabilization. Cells were then incubated with primary Ab diluted in 1% BSA/PBS at 4 degree overnight. Unbound antibody was washed out with PBS followed by incubating with diluted 2^nd^ antibody for 1 hour at room temperature. Coverslips were then mounted onto microscope glasses for visualization at ×60 magnification.

## Additional Information

**How to cite this article**: Du, W. *et al.* Hyper-active non-homologous end joining selects for synthetic lethality resistant and pathological Fanconi anemia hematopoietic stem and progenitor cells. *Sci. Rep.* **6**, 22167; doi: 10.1038/srep22167 (2016).

## Supplementary Material

Supplementary Information
