# Supplementary material for: Hyper-active non-homologous end joining selects for synthetic lethality resistant and pathological Fanconi anemia hematopoietic stem and progenitor cells
Source: Sci Rep. 2016 Feb 26;6:22167. doi: 10.1038/srep22167 (PMC4768158; doi:10.1038/srep22167)
Supplement: Supplementary Information [file srep22167-s1.pdf]

Hyper-active non-homologous end joining selects for synthetic lethality resistant and pathological Fanconi anemia hematopoietic stem and progenitor cells

Wei Du[1,2], Surya Amarachintha[1], Andrew F. Wilson[1], Qishen Pang[1,3]

[1]Division of Experimental Hematology and Cancer Biology, Cincinnati Children's Hospital Medical Center, Cincinnati, Ohio 45229, [2]Divisions of Radiation Health, College of Pharmacy, UAMS, Little Rock, Arkansas 72205, [3]Department of Pediatrics, University of Cincinnati College of Medicine, Cincinnati, Ohio 45229

Address correspondence to: Qishen Pang, Division of Experimental Hematology and Cancer Biology, Cincinnati Children's Hospital Medical Center, 3333 Burnet Avenue, Cincinnati, Ohio 45229. Phone: (513) 636-1152. Fax: (513) 636-3768. E-mail: Qishen.pang@cchmc.org or Wei Du, Division of Radiation Health, College of Pharmacy, UAMS, 4301 W Markham, Little Rock, Arkansas 72205. Phone: (501)-526-6990. E-mail: wdu@uams.edu

Table S1. Primers used for q-PCR

Name	Primer Sequence
Mouse DNA-pkcs	5-GAATTGCATTAGTGCTGTGGTGCAC-3
	5-GTACGTAGGTACTTTCCACCTGC-3
Mouse Ku70	5- AAGATTTGGACAACCCAGGCGCTAAG -3
	5-AGCTGGACGTCGCTGAAGAGGTTGGC-3
Mouse Trp53bp1	5-GAGCACAAAGCCAGTGCATA-3
	5-TGAGCATTCTCGTGAAGGTGC-3
Mouse Rad51	5-CTCATGCGTCAACCACCAG-3
	5-GCTTCAGGAAGACAGGGAGAG-3
Mouse Brca1	5 -AACCGTGTCAGAAGGCTTCC-3
	5 -AGGTTGGGTCTGCCTGTTTT-3
Mouse Brca2	5-AATGCCCCATCGATTGGTC-3
	5-AGCCCCTAAACCCCACTTCAT-3

Supplementary materials

Isolation of bone marrow cells and BM transplantation

The femora and tibiae were harvested from the mice immediately after their sacrifice with CO_2. Bone marrow (BM) cells were flushed from bones into Iscove's modified Dulbecco's medium (IMDM; Invitrogen) containing 10% FCS, using a 21-guage needle and syringe. Low-density BM mononuclear cells (LDBMMNCs) were separated by Ficoll Hypaque density gradient (Sigma-Aldrich, St. Louis, MO) and washed with IMDM medium. LDBMMNCs were depleted of lineage-committed cells using a lineage cell depletion kit (Miltenyi Biotec Inc, San Diego, CA) in accordance with the manufacturer's instruction.

For limiting dilution assay described in Figure 2A, graded numbers of test low density bone marrow cells (LDBMCs) were mixed with $2X10^5$ protector BM cells and transplanted into lethally (11 Gy) irradiated BoyJ mice ($CD45.1^+$). Donor-derived chimerism ($CD45.2^+$) was measured by flow cytometry (FACSCanto I, BD Biosciences, San Jose, CA) at 16 weeks post-BMT. For hematopoietic repopulation experiments

described in Figure 2C, 50 SLAM cells from the indicated mice along with $2X10^5$ protector cells were used for BMT. For survival experiments described in Figure 5C, 3,000 LSK cells from the indicated mice along with $2X10^5$ c-Kit-depleted protector cells were used for BMT. For serial BMT experiments described in Figures 2D and 6A, one million LDBMCs or 3,000 LSK cells from the indicated mice were transplanted into sublethally (6 Gy) irradiated primary recipients. 4 months after primary transplantation, 1-5 × 10^6 BM cells from the primary recipients were further transplanted into lethally (8 Gy) irradiated secondary recipients.

In vitro cell culture and treatment

Briefly, LSK cells were maintained in StemSpan medium supplemented with 50 ng/ml murine rTpo (Peprotech, Rocky Hill, NJ), 50 ng/ml murine rSCF (Peprotech, Rocky Hill, NJ) and 1% BSA at 37°C in normoxia (21% O_2, 5% CO_2). To examine PARP1 activation in HSPCs in response to oxidative stress, LSK cells isolated from indicated mice were treated with increasing doses of MMC (0-40 nM) or Paraquat (0-1000 μM) for the indicated time intervals followed by Flow cytometry analysis. For endogenous oxidative stress, 100 μM menadione or 10 μM PEITC were used. In experiments with anti-oxidants, 100 μM of NAC or 100 μg/ml of catalase (Sigma-Aldrich, St Louis, MO) was added to the culture for 1 h before treatment with the indicated doses of menadione or PEITC. Cell viability was determined by Trypan Blue assay. Percentages of viable cells at different time points were normalized to the number at day 0.

PARP1 activity

PARP1 activity was detected by flow cytometry as previously described[1]. Briefly, cells were fixed in 100% ethanol at 20°C for at least 20 minutes. Cells were then resuspended

in 10 mL buffer A (10mM Tris-HCl pH 7.8, 1mM EDTA [ethylenediaminetetraacetic acid], 4mM MgCl2, and 30mM 2-mercaptoethanol). After centrifugation, cells pellets were resuspended in buffer A again and transferred to a V-shaped 96-well plate on ice for at least 5 minutes. Then 20 μL of 3X reaction buffer (with or without NAD) plus 13 μL of 15mM NaCl incorporating were added to the reaction mix followed by 37°C incubation for 10 minutes. Then second fixation was done by adding 60μL of 4% formaldehyde/phosphate-buffered saline (PBS) for 20 minutes at room temperature. PBS was then added to quench the reaction. Cells were then centrifuged and resuspended in 100 μL primary PARP1 antibody (Acris Antibodies, San Diego, CA) diluted in fluorescence-activated cell sorter (FACS) buffer and incubated at 37°C for 1 hour or overnight at 4°C. Then the cells were washed and resuspended in 100 μL of diluted secondary antibody (Alexa 488-conjugated goat anti-mouse; Invitrogen) followed by 37°C incubation for 30 minutes. Cells were washed and resuspended for flow cytometry analysis.

Flow Cytometric analysis

The lineage marker (Lin) mixture (BD Biosciences, San Jose, CA) for BM cells from treated or untreated mice included the following biotinylated antibodies: CD3ε (145-2C11), CD11b (M1/70), CD45R/B220 (RA3-6B2), mouse erythroid cells Ly-76 (Ter119), Ly6G and Ly-6C (RB6-8C5). Biotinylated primary antibodies were detected by incubation of antibody-coated cells with streptavidin-PerCP or FITC (BD Biosciences, San Jose, CA) in a two-step staining procedure. Other conjugated antibodies (All from BD sciences) used for surface staining included: Sca1 (D7), c-kit (2B8), CD48 (HM48-1) and CD150 (Q38-480). Donor-derived chimerism was determined by staining peripheral blood (PB)

samples from recipient mice with antibodies specific for CD45.1 (A20), CD45.2 (A104) (BD Biosciences, San Jose, CA).

For apoptosis staining, cells were stained with Annexin V and 7AAD using BD ApoAlert Annexin V kit (BD Pharmingen, San Jose, CA) in accordance with the manufacturer's instruction. Apoptosis was analyzed by quantification of Annexin V-positive cell population by flow cytometry. For cell cycle analysis, surface markers stained cells were fixed and permeabilized with Cytofix/Cytoperm buffer (BD Pharmingen, San Jose, CA) followed by intensive wash using Perm/Wash Buffer (BD Pharmingen, San Jose, CA). Cells were incubated with anti-mouse Ki67 antibody (BD Pharmingen, San Jose, CA), washed and stained with and Propidium iodide (PI, BD Bioscience, San Jose, CA) followed by Flow cytometric analysis.

For cell sorting, lineage negative cells were enriched using lineage depletion columns (StemCell Technologies, Vancouver, BC, Canada) according to the manufacturer's instructions. The LSK (Lin^{-} $c\text{-}Kit^{+}$ $Sca\text{-}1^{+}$), HSC ($Lin^{-}ckit^{+}Sca\text{-}1^{+}CD150^{+}$ $CD48^{-}$) populations were acquired by using the FACSAria II sorter (BD Biosciences, San Jose, CA).

Molecular cloning and materials

For lentiviral vector construction, FLAG-tagged full-length PARP1 cDNA was cloned into the pRRL-SIN-cPPTMNDU3-MCS-IVW (TMND-IRES-Venus) vector (a gift from Dr. Punam Malik at the Cincinnati Children's Hospital Medical Center), which is a HIV-based self-inactivating (SIN) lentiviral vector containing the central polypurine and termination tract (cPPT).[1] The expression of PARP1 is controlled by a modified MNDU3 promoter. The vector also carries the internal ribosome entry site (IRES) followed by Venus and

woodchuck hepatitis virus post-transcriptional regulatory element (WPRE). Mutagenesis was carried out using the QuickChange Lightening Site-Directed Mutagenesis Kit (Stratagene, Santa Clara, CA). The plasmids (10 ug each) were used to produce lentiviral supernatant.

RNA Isolation, Reverse Transcriptase (RT)-PCR

Total RNA from indicated cell compartment isolated from WT mice was prepared with RNeasy kit (Qiagen, Valencia, CA) following the manufacturer's procedure. Reverse transcription was performed with random hexamers and Superscript II RT (Invitrogen, Grand Island, NY) and was carried out at 42 °C for 60 min and stopped at 95 °C for 5 min. First-strand cDNA was used for real-time PCR using primers listed in Table S1. Samples were normalized to the level of *GAPDH* mRNA.

Statistical analysis

Paired or unpaired student's *t-test* was used for two-group comparisons. Survival data were plotted by the Kaplan-Meier curve method and analyzed by the log-rank test. Values of $p<0.05$ were considered statistically significant. Results are presented as mean ± SD. * indicates $p<0.05$; ** indicates $p<0.01$; *** indicates $p<0.001$.

References:

1. Li, X., Erden, O., Li, L., Ye, Q., Wilson, A. & Du W. Binding to WGR domain by salidroside activates PARP1 and protects hematopoietic stem cells from oxidative stress. *Antioxid Redox Signal* 20(12): 1853-1865 (2014).
2. Wang, J., Sun, Q., Morita, Y., Jiang, H., Gross, A., Lechel, A. *et al.* A differentiation checkpoint limits hematopoietic stem cell self-renewal in response to DNA damage. *Cell* 148(5): 1001-1014 (2012).

Supplementary Figures

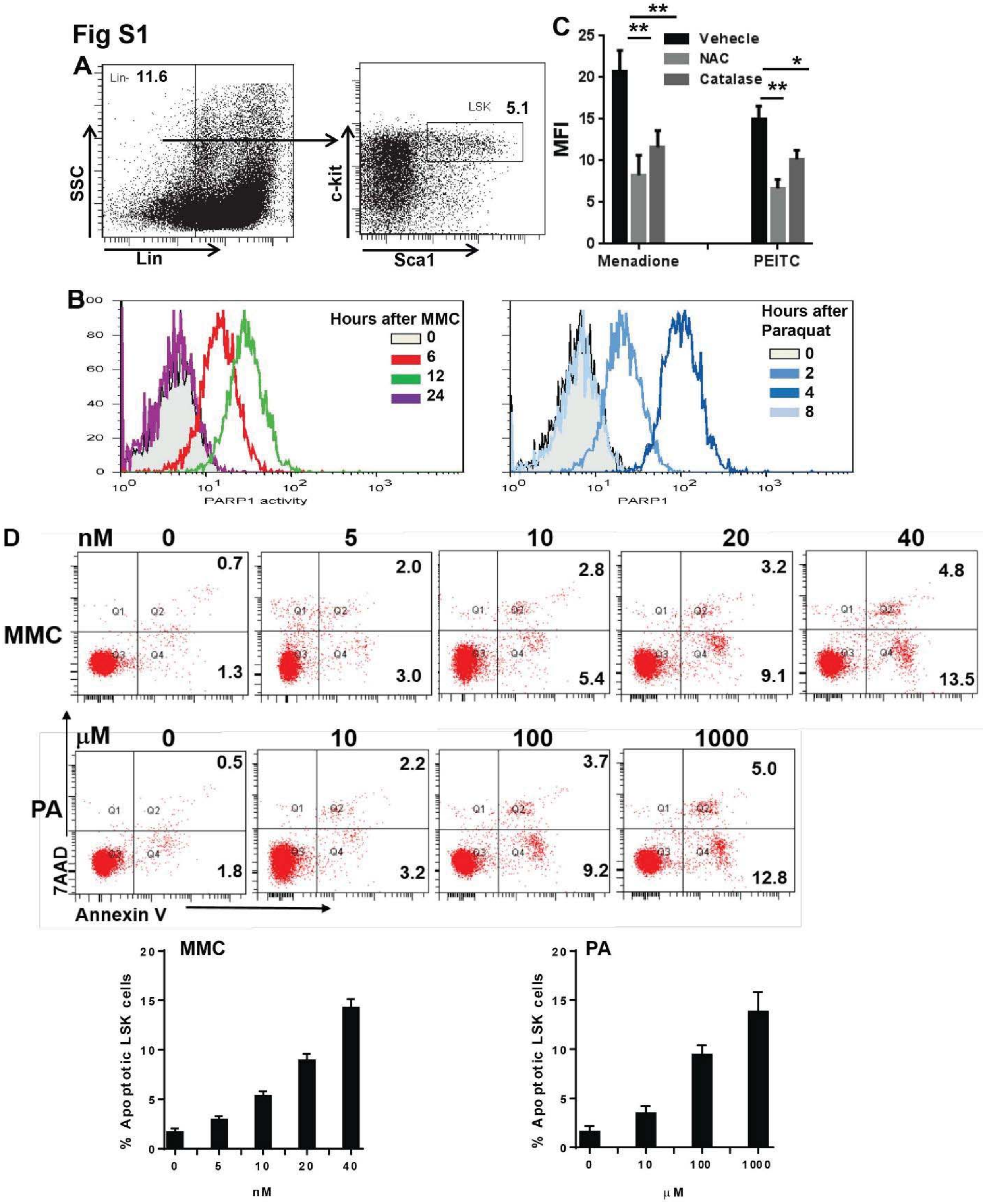

Fig S1. PARP1 activation in HSPCs in response to genotoxic and oxidative stress.

(A) Gating strategy for isolation of BM LSK ($Lin^{-}c\text{-}kit^{+}Sca\text{-}1^{+}$) cells from WT C57BL/6 mice. (B) Kinetics of PARP1 activation in response to MMC or Paraquat. LSK cells from WT mice were cultured *in vitro* in the presence of MMC (20 nM; Left) or Paraqut (100 μM; Right) for the indicated time intervals followed by Flow cytometric analysis for Parp1 activity. (C) Antioxidants reduce PARP1 activity. Cells described in (B) were pretreated with NAC (100 μM) or catalase (100 μg/ml) for 1 h followed by *in vitro* culture in the presence of 100 μM menadione or 10 μM PEITC. Cells were then subjected to Flow Cytometric analysis for PARP1 activity. Quantification of MFI was shown. Results are means ± standard deviation (SD) of 3 independent experiments (n=6 per group). (D) Apoptosis induced by genotoxic and oxidative stresses. LSK cells from WT mice were cultured *in vitro* in the presence of the indicated doses of MMC (0-40 nM) for 12 h or Paraquat (0-1000 μM) for 4 h followed by Annexin V/7AAD staining for apoptosis analysis. Representative flow plots (Upper) and quantification (Lower) are shown.

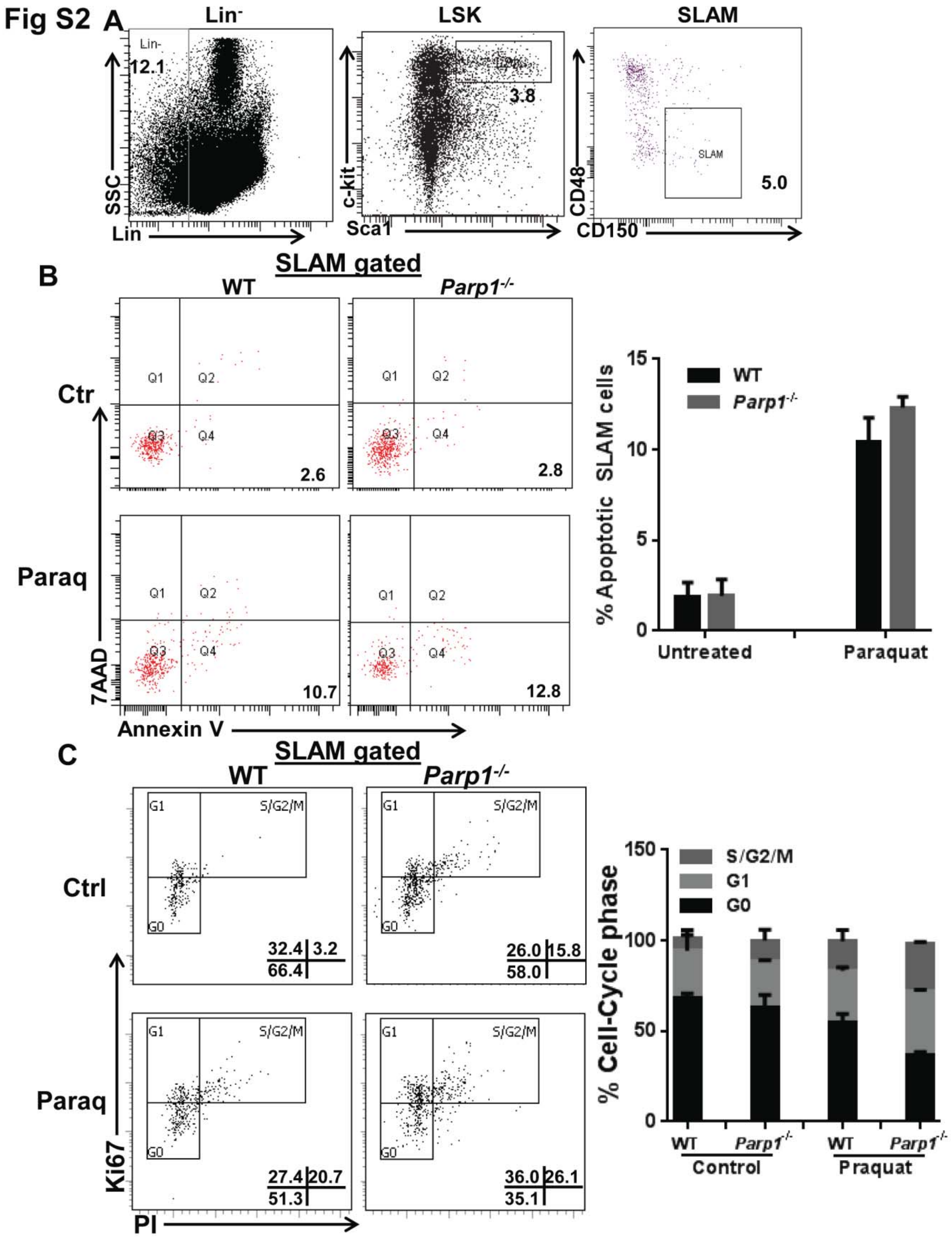

Fig S2. PARP1 activation attenuates Oxidative stress-induced HSC exhaustion. (A) Gating strategy for Flow Cytometric analysis. (B) Oxidative stress does not affect

apoptosis in HSCs *in vivo*. Single dose (10 mg/kg) of paraquat was i.p. injected to WT or *Parp1*$^{-/-}$ mice. LDBMCs were then isolated and analyzed for apoptosis in SLAM cells by Annexin V/7AAD staining. Representative dot plots and quantification are shown. (C) Oxidative stress induces cell cycling in HSCs *in vivo*. Single dose (10 mg/kg) of paraquat was i.p. injected to WT or *Parp1*$^{-/-}$ mice. LDBMCs were then isolated and analyzed for cell cycle status in SLAM cells by Ki67/PI staining. Representative dot plots and quantification are shown.

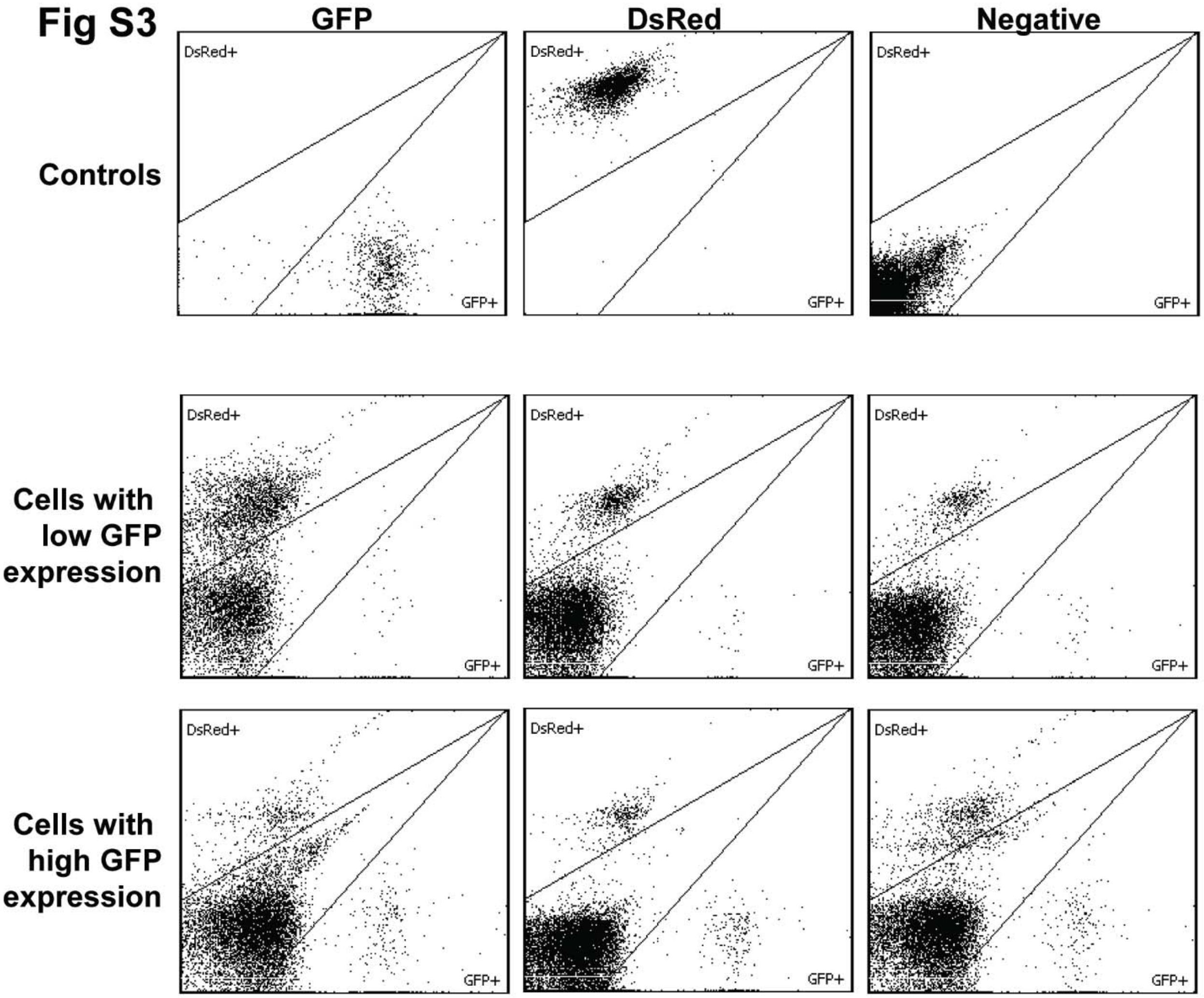

Fig S3. Calibration of the parameters for FACS analysis in eGFP-based reporter

assay. Cells were analyzed on a red-versus-green fluorescence plot. The gating for the analysis of green and red cells was set up by using cells transfected with 0.5 μg of GFP or 0.1 μg of DsRed vectors. GFP and DsRed cells possess autofluorescence and fall along the green red diagonal, whereas GFP+ and DsRed+ cells appear in separate populations shifted off the diagonal.

Fig S4

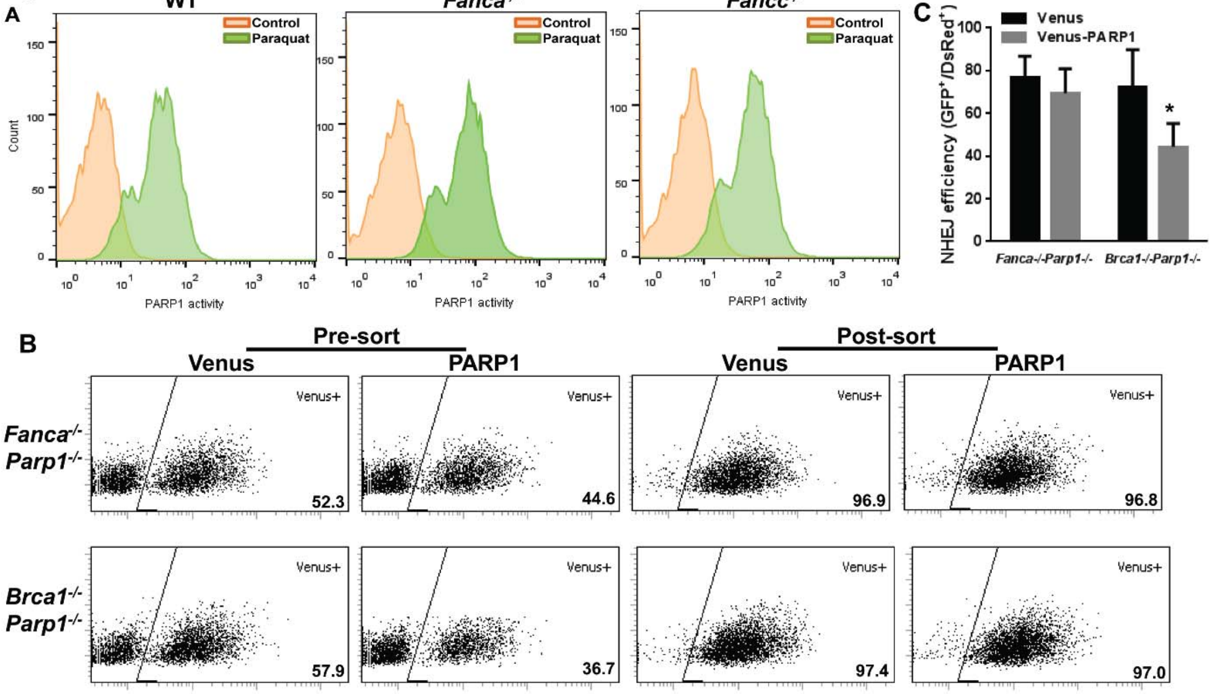

Fig S4. Suppression of NHEJ by PARP1 requires FA core complex components Fanca and Fancc. (A) Oxidative stress-induced PARP1 activation is preserved in FA deficient HSPCs. LDBMCs from WT, *Fanca-/-* or *Fancc-/-* mice were treated with Paraqut (100 μM) for 4 hours followed by Flow Cytometric analysis for PARP1 activity in LSK cells. (B) Transduction efficiency of lentiviral vector expressing Venus or Venus-PARP1. LSK cells isolated from *Fanca-/-Parp1-/-* or *Brca1-/-Parp1-/-* DKO mice were transduced with lentiviral vectors expressing Venus or Venus-PARP1. Representative dot plots of pre- or

post-cell sort are shown. (C) Re-expression of PARP1 partially rescues the hyper-active NHEJ phenotype in *Brca1*$^{-/-}$*Parp1*$^{-/-}$ but not in *Fanca*$^{-/-}$*Parp1*$^{-/-}$ DKO cells. Mouse embryonic fibroblasts (MEFs) isolated from *Fanca*$^{-/-}$*Parp1*$^{-/-}$ or *Brca1*$^{-/-}$*Parp1*$^{-/-}$ DKO mice were transduced with lentiviral vectors expressing Venus or Venus-PARP1. Sorted Venus^{+} cells were transfected with plasmids expressing NHEJ-eGFP and DsRed followed by *in vitro* culture with MMC (20 nM) for 12 hours. Repair efficiency was determined by the ratio of eGFP to DsRed.
